# Correction: Measuring psychological capital: Revision of the Compound Psychological Capital Scale (CPC-12)

**DOI:** 10.1371/journal.pone.0301794

**Published:** 2024-04-02

**Authors:** Ludmila Dudasova, Jakub Prochazka, Martin Vaculik, Timo Lorenz

In the Study 3 subsection of the Results, there is an error in the items of new resilience in the fourth paragraph. The correct new resilience items are:

- I consider myself a person who can withstand a lot.
- Failure does not discourage me.
- I tend to bounce back quickly after serious life difficulties.
