# Supplementary material for: Measuring psychological capital: Revision of the Compound Psychological Capital Scale (CPC-12)
Source: PLoS One. 2021 Mar 3;16(3):e0247114. doi: 10.1371/journal.pone.0247114 (PMC8508554; doi:10.1371/journal.pone.0247114)
Supplement: S1 Appendix — (DOCX) [file pone.0247114.s005.docx]

**CPC-12R in Czech**

*1 = silně nesouhlasím, 2 = nesouhlasím, 3 = spíše nesouhlasím, 4 = spíše souhlasím,*

*5 = souhlasím, 6 = silně souhlasím*

1. Těším se na život, který mám před sebou.

2. Očekávám, že se mi stane více dobrých než špatných věcí.

3. Věřím, že mi budoucnost přinese mnoho dobrého.

4. Pokud bych se ocitl/a v nesnázích, dokázal/a bych přijít na více způsobů, jak se z nich dostat.

5. Napadá mě mnoho způsobů, jak dosáhnout svých cílů.

6. V této chvíli se pokládám za poměrně úspěšného/úspěšnou.

7. Věřím, že si dokážu s neočekávanými událostmi poradit.

8. Pokud investuji potřebné úsilí, zvládnu vyřešit většinu problémů, se kterými se setkám.

9. Dokážu zachovat klid i v obtížných situacích, protože se mohu spolehnout, že je zvládnu.

10. Považuji se za člověka, který hodně vydrží.

11. Neúspěch mě neodradí.

12. Po vážných životních těžkostech se dám zase rychle do pořádku.

**CPC-12R in English**

*1 = strongly disagree, 2 = disagree, 3 = slightly disagree, 4 = slightly agree,*

*5 = agree, 6 = strongly agree*

1. I am looking forward to the life ahead of me.
2. Overall, I expect more good things to happen to me than bad.
3. The future holds a lot of good in store for me.
4. If I should find myself in a jam, I could think of many ways to get out of it.
5. I can think of many ways to reach my current goals.
6. Right now, I see myself as being pretty successful.
7. I am confident that I could deal efficiently with unexpected events.
8. I can solve most problems if I invest the necessary effort.
9. I can remain calm when facing difficulties because I can rely on my coping abilities.
10. I consider myself a person who can withstand a lot.
11. Failure does not discourage me.
12. I tend to bounce back quickly after serious life difficulties.
