# Supplementary material for: Measuring psychological capital: Revision of the Compound Psychological Capital Scale (CPC-12)
Source: PLoS One. 2021 Mar 3;16(3):e0247114. doi: 10.1371/journal.pone.0247114 (PMC8508554; doi:10.1371/journal.pone.0247114)
Supplement: Attachment — Submitted filename: Response to Reviewers.docx [file pone.0247114.s006.docx]

*Dear editor and reviewers,*

*We would like to thank you for all the valuable comments and for revising our manuscript. We have addressed and incorporated them in the revised version, as described below. The changed sentences are highlighted. We believe that the changes helped in strengthening our paper.*

*I would also like to inform you that I got married recently and my family name has changed from Kašpárková to Dudášová.*

*Sincerely,*

*Ludmila Dudášová*

Responses to reviewers’ comments:

**Reviewer 1:**

Response: *We want to thank Reviewer 1 for their positive evaluation of our study and for the following comment that helped us in improving our manuscript.*

For both exploratory factor analysis (EFA) and confirmative factor analysis (CFA), the matric of factor loadings is very useful information besides fitness indices of models，such that I suggest the authors should present specific factor loadings or factor measurement model of CFA to help reader understanding.

Response: *Thank you for your remark. We share the conviction that factor loadings represent a very useful information alongside fitness indices of the models.*

*Responding to feedback from the second reviewer, we decided to remove EFA from the manuscript.*

*The purpose of the EFA was to show that the data cannot be better explained by any other model than one of the models derived from the theory. However, we agree with the second reviewer that EFA is not necessary to meet the objectives of the study, and that CFAs provide all the information needed to test our hypotheses. Nevertheless, if you find it relevant, we are prepared to insert it back, and to add a table with factor loadings.*

*In relation to CFA, we have added new tables with factor loadings in Study 1 (Table 2, p. 9) and Study 2 (Table 6, page 15; Table 7, p. 16). In Study 2, specific factor loadings have been presented for models with three and four first-order factors. In Study 3, the factor loadings are available in the Figure.*

**Reviewer #2:**

Response: *We want to thank Reviewer 2 for reading our manuscript carefully and for providing their valuable comments. They helped us in making the aim and contributions of our manuscript clearer and in adding the missing information important for understanding the procedures and analyses.*

The study " Measuring Positive Psychological Capital: Revision of the CPC-12" is clear and well-written. I found the purpose of the study very practical. Yet, I have more concerns about the hypotheses, the analysis method used and the theoretical background of the paper. Such major concerns should be further taken into consideration in this paper.

1. According to the title and the part of Materials and Methods, the target construct was positive psychological capital, but in the introduction part, only the construct of psychological capital was mentioned. The relation of positive psychological capital and psychological capital should be provided or the expression should be consistent.

Response: *Thank you carefully reading our text. We omitted the word “positive” in the first version of the manuscript. Thanks to your comment, we questioned whether the word indeed has a function and concluded that we can continue using the term “psychological capital” (as used in the original article introducing CPC-12 scale). Therefore, we replaced the terms “positive psychological capital” with “psychological capital.”*

2. The most significant problem I found was that the hypotheses of the current study was unclear, which made the study design data-driven. As I understand, the central purpose was to develop a Czech version of Compound Psychological Capital Scale. In the first study, the results showed that the construct validity was not confirmed. To this end, the authors should consider modifying the items or discussing other constructs of psychological capital. I didn’t find much need to do the secondary analysis of the original data of Lorenz et al, because I didn’t see the hypotheses to guide the analysis.

Response: *Thank you for your suggestion. We recognize the need to clarify the rationale of our research. Our original goal was to adapt a Czech version of the questionnaire. With this intention, we designed and executed the first study. However, based on the analysis of the newly collected data, the goal of the study changed and now the aim of the manuscript is to draw attention to the significant limitations of the published questionnaire CPC-12 and to propose a revised version. Please see the second paragraph on p. 6, where we explain the logic of our study.*

*None of the studies were data-driven. In the first study, we began with the hypothesis that the data obtained by the Czech adaptation of CPC-12 would correspond to the theoretical four-factor model of positive psychological capital (p. 6, third paragraph). However, we did not find support for this hypothesis. Consequently, we analyzed the data and searched for an explanation for this result. We found out that items from the resilience and self-efficacy subscales tended to load on a single common factor.* *Initially, we focused on the content of the Czech items, believing that inaccurate translation had led to such results. However, we concluded that the multi-stage translation was performed well, and the content of the Czech items corresponded to the content of the original items.*

*Therefore, we decided to inspect the data from the original German study and hypothesized that we would find the same problem with the factor structure (p. 12, lines 265-269). In Study 2, this hypothesis was proven correct; we found the same problem which provided important evidence about limitation of the original scale. We consider this to be a very important finding as it shows that the article previously published in PLOS ONE does not contain precise conclusions, and that CPC-12 does not measure positive psychological capital as a construct with four first-order factors. The aim was now to inspect the possible cause of the findings. There were three possible causes to investigate (p. 10): translation error, an error in PsyCap theory and limitations of the original CPC-12. Upon further analysis, we found that the items intended to measure resilience do not match the content of the resilience construct. This conclusion (low factor loadings of resilience items, low reliability of the resilience subscale) (p. 17, second paragraph) led to study 3.*

*Consequently, we proposed new items for measuring resilience so that they better match the content of the construct and that the CPC-12R scale measures all 4 components of positive psychological capital. In the third study, we tested whether the data obtained using CPC-12R fit the theoretical 4+1 factor model and also if the 4+1 model of PsyCap explained the data better than the more parsimonious model with 3+1 factors which was preferred when using the original scale (p. 17, third paragraph). We found support for these hypotheses and provided evidence on factorial validity and reliability of the revised scale. We also suggested directions for further validation of the revised questionnaire.*

*We believe that all three studies are important for our article. The first study explains our motivation to revise the scale and provide independent evidence about its psychometric limitations. The second study draws attention to the problem in the interpretation of data in the study previously published by Lorenz et al. (2016) and to the shortcomings of the published scale (CPC-12). The third study proposes a new version of the scale (CPC-12R) which deals with the previously mentioned limitations and provides initial evidence on factorial validity and reliability of the revised scale.*

*We agree that the hypotheses were not evident from the text of the manuscript. Thanks to your feedback, we have now explicitly stated the hypotheses for all three studies.*

3. The other issue in the current study was that the theoretical background was limited. In the introduction part, except for PCQ and CPC-12, not much information was given on the measurement of psychological capital. How the dimensions of psychological capital were defined by other studies? Is the second-order four-factor construct supported in other studies? How the self-efficacy and resilience related? The authors should provide more evidence to support your study. More important, positive and negative evidence about the validity of CPC-12 should be provided, considering the current study was based on the adaptation of CPC-12. Those evidence could be the foundation of subsequent analysis of results.

Response: *Thank you for your suggestion to implement more details about PsyCap measurement. Please see pp. 4-5, where we provide additional information. Other studies define the dimensions of PsyCap comparably to how we defined it (see, e. g. Luthans, Avey and Patera, 2008, listed as citation number 5 in our manuscript) and the second-order four-factor construct is supported by other studies. Reader can find this information on p. 2, lines 42-43 (“The existence of a higher order core construct, PsyCap, has both conceptual [1] and empirical [5] support.”).*

*To help reader understand the relationship between resilience and self-efficacy, we start by providing detailed definitions of the constructs. We note that these constructs are close, hence we describe the shared content as well as the difference between them. According to research that has examined the relationship between these variables, the relationship between them is significant, but not so strong that we doubt that they are two different constructs. Please see pp. 10-11 (lines 223-234).*

*When we found the overlap between them, we examined carefully both the items measuring resilience and self-efficacy.* *We concluded that while items intended to measure self-efficacy are in line with the definition of self-efficacy, items intended to measure resilience do not reflect the definition of resilience. This led us to keep the items measuring self-efficacy and replace the items measuring resilience. Thanks to your suggestion, we describe it now in the manuscript more clearly (see pp. 17-18, lines 353-374).*

*As for the positive and negative evidence about the validity of CPC-12, the number of published studies examining the psychometric properties of the scale remain very limited as the scale was published recently. However, in response to your comment, we addressed this on p. 5 (see changes on lines 111-113).*

4. I have some confusion about the analysis method used. In three studies, MLR was used as in the study of Lorenz et al. However, estimation was determined mainly by the data pattern. How it’s verified that MLR was the most appropriate estimator? Also, it’s unclear why Varimax rotation was used, instead of oblique rotation method.

Response: *Because items had a sufficiently large response scale to be approximated as an interval variable, we used maximum likelihood estimation instead of estimator that assumes ordinal nature of the data. Due to the rather small sample size, results using the maximum likelihood robust estimation are less likely to be biased than results using WLSMV or similar estimator ( for details see Rhemtulla et al., 2012, Sass et al., 2014 and Li, 2016 ). We believe that the use of a robust maximum likelihood estimator for CFA of scales with a five-point response scale is so widespread that it does not need to be specifically justified. It is also noteworthy that the original study did not mention the estimator and it is not common to justify the estimator on PLOS ONE manuscripts. However, if the reviewer wishes, we can add the above-mentioned explanation to the manuscript.*

*We used the predefined Varimax rotation for the EFA. We agree that the oblique rotation would be more appropriate given that the model assumes highly correlated factors. We recalculated the results using GEOMIN (OBLIQUE) rotation and the outputs are comparable to the original outputs with Varimax rotation. However, according to one of your comments (mentioned ahead) we have removed the EFA from the manuscript as the analysis did not contribute significantly to the aim of the manuscript (see below), but we are ready to insert it again if so advised.*

*References:*

Li, C. H. (2016). Confirmatory factor analysis with ordinal data: Comparing robust maximum likelihood and diagonally weighted least squares. *Behavior research methods*, *48*(3), 936-949. <https://doi.org/10.3758/s13428-015-0619-7>

Rhemtulla, M., Brosseau-Liard, P. É., & Savalei, V. (2012). When can categorical variables be treated as continuous? A comparison of robust continuous and categorical SEM estimation methods under suboptimal conditions. *Psychological methods*, *17*(3), 354-373. [https://doi.org/10.1037/a0029315](https://psycnet.apa.org/doi/10.1037/a0029315)

Sass, D. A., Schmitt, T. A., & Marsh, H. W. (2014). Evaluating model fit with ordered categorical data within a measurement invariance framework: A comparison of estimators. *Structural Equation Modeling: A Multidisciplinary Journal*, *21*(2), 167-180.

https://doi.org/10.1080/10705511.2014.882658

5. In the results part, more important information should be added, including the descriptive statistics, reliability coefficients, and the full factor loadings in each study. The results mainly focused on the model fit, while it’s also essential to analyze how items were distributed to different factors. For example, in study 1, it said that “Although there were a number of cross-loadings , items from the hope subscale tended to load on one common factor, ……”, how the items were distributed should also be analyzed except for the model fit results. Besides, the 3-factor model was not significantly better fitted than 4-factor model. It’s not adequate to conclude that “three-factor model of PsyCap fit the data better”.

Response: *We have added tables containing descriptive statistics, reliability coefficients, and factor loadings for all studies to the manuscript.*

*Thank you for pointing out the misinterpretation of the comparison of the two models. We have corrected the text (see changes on p. 8).*

6. In the discussion part of study 1, the authors provided three assumptions to explain the results. Which assumption did the authors support, according to the theoretical evidence and the results? How did the previous studies find the relations between self-efficacy and resilience? The discussion lacked evidence to support the opinions.

Response: *Thank you for your valuable comment. We have made significant changes in the discussion of study 1 to help reader understand our decisions (please see changes on pp. 9-12).*

*The relationship between resilience and self-efficacy had already been investigated. We now added that the previous studies found these two concepts to be moderately to strongly related. However, the studies also showed that resilience and self-efficacy are correlated but different and not linearly dependent constructs, and that factor analysis (using PCQ) is able to distinguish among them. Our finding that resilience and self-efficacy cannot be distinguished are not in line with previous studies. We hope that it is now more evident from the text of the manuscript.*

7. According to the analysis of study 1, in Lorenz’s study, the model included “12 items, four first-order factors (i.e., hope, self-efficacy, optimism, and resilience), and one second-order factor”. Why the models in study 2 were all one-order models in EFA? (“the analyses on both datasets identified one, two, and three-factor solutions”). What is the purpose of EFA? Besides, according to the results in Table 4, the difference between 3-factor and 4-factor model was not significant.

Response: *Thank you for raising this issue. It is not possible to perform EFA with a second-order factor. For second-order factor models, multiple EFAs are made, one for each second-order factor. Because all four components of PPC are saturated with the same second-order factor, we consider our approach to be correct.*

*The purpose of the EFA was to show that the data cannot be better explained by any other model than one of the models derived from the theory. However, we agree that EFA is not necessary to meet the objectives of the study and it is not appropriate for the study that does not want to be exploratory and data driven. CFAs provide all the information needed to test our hypotheses. Hence, we have removed the EFA from the manuscript. However, we are ready to insert it again if you so recommend.*

8. In study 3, why items on resilience were modify instead of self-efficacy? To adapt one subscale, only four new items were prepared. It was not adequate. Additionally, How the removed items were determined? According to the article, “To limit possibility of bias caused by the order of items, we placed the fourth reserve item measuring resilience at the end of the questionnaire.” How did the author know that the fourth item will be removed in advance?

Response: *We replaced the resilience items because their original content did not accurately reflect the content of the resilience construct. This is not the case with the self-efficacy subscale; items in it adequately reflect the self-efficacy construct. We emphasized this explanation in the text of the study to make it clear (pp. 17-18). We now also show more precisely the empirical evidence of the low content validity of the original resilience subscale.*

*While we created several new items, we chose the three items which we thought described the content of the resilience construct well. These items covered the whole construct of resilience (as it is defined as a part of the PsyCap definition). We chose the fourth spare item and added it in the end of the questionnaire in case the analysis revealed a problem in one of the first three proposed items. However, as the first three items worked as expected, we left the spare item from further analyses. We wanted the revised version to have the same number of items as the original version, and for all subscales to have the same number of items.*

*We agree that including only four items in the data collection could represent a risk if more than one item showed to be problematic. However, this has not happened.*

9. In discussion of study 3, it said that “There was a difference in the ratio of items measuring individual components compared to PCQ-12”. However, how items were modified was not introduced in advance, which caused much confusion.

Response: *Thank you for your valuable comment. We agree that this part of the text was confusing. We did not change the number of items measuring each component. We only wanted to inform the readers that CPC-12 measures PsyCap by 3+3+3+3 items, and therefore the effects of all components are balanced, which is a benefit compared to PCQ-12 which is composed of 3+4+3+2 items. Following your comment, we removed this information from the discussion of study 3 and inserted it in the introduction of study 1, where we introduce and compare existing scales (p. 5, first paragraph).*

10. For a study to develop scales, reliability evidence and more validity evidence should be provided. Though it was mentioned in the limitations, it remained a fatal flaw.

Response*: Thank you for raising this concern. The main contribution of our manuscript is not the new scale, but the fact, that we found a psychometric limitation of the original scale that was published in PLOS ONE. However, we did not just want to find limitations and leave it at that. We also wanted to show that the problem can be solved by replacing items that were used for measuring resilience. The contribution of Study 3 is not only in providing the revised scale, but also in showing that we found the correct cause of the problem that we identified in Study 1 and Study 2 and that the problem can be solved.*

*If the aim was to develop a new scale, we would arrange Study 1 as a study focused on developing and choosing the best items and we would add further studies to provide evidence about the validity of the new scale. However, our aim was different. We presented two studies that highlighted the problem in a published and widely cited scale and we arranged the third study to suggest the solution. We agree that further studies are needed to provide more evidence on the validity of the revised scale, and we state it in the general discussion (see p. 24, lines 507-510).*
